# Understanding of a negative bowel screening result and potential impact on future symptom appraisal and help‐seeking behaviour: a focus group study

**DOI:** 10.1111/hex.12484

**Published:** 2016-07-14

**Authors:** Karen N. Barnett, David Weller, Steve Smith, Sheina Orbell, Peter Vedsted, Robert J. C. Steele, Jane W. Melia, Sue M. Moss, Julietta Patnick, Christine Campbell

**Affiliations:** ^1^ University of Edinburgh Edinburgh UK; ^2^ Midlands and NW Bowel Cancer Screening Programme Hub UHCW NHS Trust Rugby UK; ^3^ University of Essex Colchester UK; ^4^ University of Aarhus Aarhus Denmark; ^5^ University of Dundee Dundee UK; ^6^ University of Cambridge Cambridge UK; ^7^ Queen Mary University of London London UK; ^8^ University of Oxford Oxford UK

**Keywords:** bowel cancer, colorectal cancer, colorectal cancer screening, focus groups, guaiac faecal occult blood test, health knowledge, negative screening results, Scotland, symptom appraisal

## Abstract

**Background:**

Colorectal cancer (CRC) screening using a faecal occult blood test (FOBt) has the potential to reduce cancer‐related mortality. Symptom vigilance remains crucial as a proportion of cancers will be diagnosed between screening rounds. A negative FOBt has the potential to influence how participants respond to future symptoms of CRC.

**Objective:**

To explore (i) understanding of a negative FOBt and (ii) the potential impact of a negative FOBt upon future symptom appraisal and help‐seeking behaviour.

**Design:**

Qualitative methodology utilizing focus groups with participants who received a negative FOBt within the National Bowel Cancer Screening Programme in Coventry and Lothian. Topics explored included: experience of screening participation, interpretation and understanding of a negative result, symptom awareness and attitudes towards help‐seeking.

**Results:**

Four broad themes were identified: (i) emotional response to a negative FOBt, (ii) understanding the limitations of FOBt screening, (iii) symptom knowledge and interpretation and (iv) over‐reassurance from a negative FOBt. Participants were reassured by a negative FOBt, but there was variability in the extent to which the result was interpreted as an “all clear”. Some participants acknowledged the residual risk of cancer and the temporal characteristic of the result, while others were surprised that the result was not a guarantee that they did not have cancer.

**Discussion and conclusions:**

Participants recognized that reassurance from a negative FOBt could lead to a short‐term delay in help‐seeking if symptoms developed. Screening programmes should seek to emphasize the importance of the temporal nature of FOBt results with key messages about symptom recognition and prompt help‐seeking behaviour.

## 1 Introduction

In the United Kingdom, colorectal cancer (CRC) is the fourth most common cancer and is the second most common cause of cancer death.^1^, ^2^ There is a growing body of evidence that early diagnosis of CRC leads to more favourable outcomes,^3^, ^4^, ^5^, ^6^ with a number of countries introducing CRC screening programmes for the general/average risk population. Biennial CRC screening using a guaiac faecal occult blood test (gFOBt) was first rolled out nationally in Scotland in 2006, with complete coverage by 2010, and similar programmes were introduced across the rest of the United Kingdom at the same time. A positive test result indicates that further investigation (usually a colonoscopy) is required.^7^ The most recent Cochrane review on CRC screening using gFOBt suggests a 15% relative risk reduction in CRC mortality for trials that used biennial screening.^8^ In the United Kingdom the majority of the screened population receive a negative gFOBt result, while approximately 2% of the population receive a positive result fewer than 10% of these (0.2%) are attributable to CRC.^9^, ^10^

Some CRCs do not lose sufficient blood to be detected by a gFOBt which can result in low sensitivity,^11^ reported to be down to 0.55 in randomized controlled trials (RCTs).^8^ Pilot screening programmes and trials in the United Kingdom have reported a false‐negative proportion between 30% and 50%.^9^, ^12^, ^13^

Interval cancers are defined as “CRC diagnosed after a screening or surveillance episode in which no cancer is detected, and before the date of the next recommended test”.^14^, ^15^ Thus, CRC screening using gFOBt misses a proportion of cancers, and cancers can develop between screening rounds—even with a fully implemented screening programme up to 75% of all CRC's will be diagnosed following symptomatic presentation.^16^ It is therefore important that screening programmes run alongside diagnostic pathways, particularly in general practice, to ensure early symptomatic presentation and timely referral.

Participation in screening has the potential to influence how individuals respond to bowel symptoms, but to date, there is little evidence to suggest how a negative test result might influence symptom appraisal and help‐seeking behaviour. A recent UK study of patients with symptoms suggestive of CRC found that a recent negative screening test result was perceived to be reassuring, particularly if cancer had been considered as a possible cause of the experienced symptoms.^17^ In cervical cancer screening, one study found that only 52% of women correctly understood that a “normal” test result entailed a residual risk of cervical cancer.^18^ Solbjør et al. (2012)^19^ reported that some women who experienced possible symptoms of breast cancer delayed help‐seeking if symptoms occurred following a recent negative mammography scan because they assumed that their palpable lesion would be benign.

In summary, to date, there is a paucity of research investigating understanding of a negative gFOBt result among bowel screening participants. The purpose of this study was to explore (i) how a negative gFOBt result is understood by screened participants and (ii) in what ways a negative gFOBt result might impact upon future symptom appraisal and help‐seeking behaviour if symptoms develop.

## 2 Methods

### 2.1 Study design

A focus group study with individuals who had participated in the bowel cancer screening programmes in Scotland (Lothian) and England (Coventry) within the last 4 months and whose result was negative. Focus groups were chosen to allow for the exploration of a broad and diverse range of viewpoints and to encourage discussion between participants to help draw out any latent issues.

### 2.2 GP practice recruitment

Focus group participants were recruited via their general practice on behalf of the research team. Two practices were recruited in Lothian, Scotland, and five in Coventry, England. Practices were recruited via the National Institute for Health Research (NIHR) Clinical Research Network (England) or established research links (Scotland). Practices were purposively selected to include varied locations, for example both inner and outer city practices and a mix of deprived and affluent postcodes. Practice selection was informed by publically available information on practice demographics (Information Services Division, Scotland, and Public Health England websites) and advice from the local primary care research team (Lothian) and NIHR (Coventry) who had a local knowledge of the geographical area and were familiar with the practice populations served by each practice. A modest monetary reimbursement was offered to practices in exchange for assisting with study recruitment.

### 2.3 Participant recruitment

Practice staff generated a list of approximately 60 individuals who had participated in bowel screening and received a negative gFOBt result within the previous 4 months (extended to 6 months for practices with a smaller list size). Sixty individuals were selected based on a 10%–15% response rate achieved in a similar study, led by Weller et al., University of Edinburgh, that recruited healthy participants in this age group via general practices to participate in a focus group—manuscript in preparation. Practice staff were responsible for patient identification based on the inclusion/exclusion criteria provided by the research team. These individuals were sent an invitation letter from the GP practice on behalf of the research team including an information leaflet, response form, consent form and a pre‐paid return envelope. Based on the literature and prior experience in focus group research, the research team anticipated that between six and eight focus groups (comprising of six to eight individuals) would be sufficient to provide a range of views and reach theoretical saturation.^20^

Once a response form was returned, participants were contacted by the researcher to confirm a time and date for the focus group to take place. Focus groups were held on the general practice premises. At the end of each focus group, participants were given an information leaflet about CRC and offered a £15 high street voucher to thank them for their contribution.

### 2.4 Exclusion criteria

Practice staff were asked to exclude potential participants if they had a recent bereavement, a recent cancer diagnosis (any) or were currently undergoing any major treatments.

### 2.5 Focus groups

Focus groups were carried out between July 2014 and November 2014. Focus group discussions lasted between 60–90 minutes. Signed consent was obtained from each participant. Focus groups were audio‐recorded, professionally transcribed verbatim and anonymized. Audio files were deleted once the written transcripts had been received and verified by the researcher. Topics explored in the focus group included the following: experience of screening participation, interpretation of a negative result, understanding of the limitations of the gFOB test, symptom awareness and attitudes towards help‐seeking.

### 2.6 Ethical review

The project was granted ethical approval from the South East Scotland Research Ethics Committee 01 (11/SS/0006), on 26 August 2011.

### 2.7 Patient and public involvement and engagement

Patient and public involvement and engagement in this project came through discussion of the design and interpretation of results with lay members of the South East of Scotland Primary Care (SCAN) Network.

### 2.8 Analysis

Thematic analysis of the data was undertaken (KB) using NVivo qualitative data analysis Software; QSR International Pty Ltd. Version 10, 2012 and was on‐going throughout the study to allow emerging themes to be fed back into the data collection. An inductive reasoning approach was adopted where themes (or categories) were identified through careful examination and comparison of the data.^21^ To ensure inter‐rater reliability, a second member of the research team (CC) read all focus group transcripts and independently coded two transcripts, emerging issues were discussed, and a coding frame agreed. Additional codes were added to the coding frame as appropriate. Final themes were agreed through an iterative process involving the core and wider research group. The analysis focused on the main themes surrounding the interpretation and influence of a negative gFOBt result on future help‐seeking behaviour.

## 3 Results

### 3.1 Recruitment

Six focus group were carried out, two in Lothian, Scotland, and four in Coventry, England. Between 3 and 10 participants attended each focus group, with a total of 35 participants (16 male) taking part. A summary of participant demographics and demographics of the recruiting general practices in Lothian and Coventry are provided in Table 1. Core themes were common to all of the focus groups, and no new themes were emergent as the sixth focus group was analysed.

**Table 1:** Population demographics: focus group participants and recruiting general practices

Focus group (location)	N (Male)	Age range (self‐report: 5‐year age categories)	Previous cancer diagnosis (N) (self‐report)
Participant demographics			
1 (Coventry, England)	6 (3)	60–74	0
2 (Coventry, England)	3 (1)	60–69	1 (cervical)
3 (Lothian, Scotland)	6 (3)	50–74	1 (breast)
4 (Coventry, England)	6 (3)	60–69	2 (breast)
5 (Coventry, England)	10 (4)	60–74	1 (skin)
6 (Lothian, Scotland)	4 (2)	55–74	0

GP practice (FGs)	Practice list size	Location type	Deprivation indicator
General practice demographics (serving invited population)^36^, ^37^			
England			Deprivation Deciles (1 = most deprived; 10 = least deprived)
Coventry (1 & 2)	10 912	Small Urban Area	4
Coventry (1 & 2)	8269	Outer City	2
Coventry (1 & 2)	8860	Inner City	6
Coventry (4)	5142	Outer Suburb	6
Coventry (5)	15 162	Outer Suburb	5
Scotland			% Practice population living in data zones defined as the 15% most deprived
Lothian (3)	11 520	Small Urban Area	<5
Lothian (6)	13 020	Inner City	43.2

### 3.2 Findings

The majority of participants who attended a focus group had engaged in more than one round of CRC screening with high experience of the the bowel cancer screening programmes. Across all six focus groups, screening was viewed positively among the participants with general agreement on the importance, or benefits, of the early detection of cancer with some acknowledging the importance of continued participation in cancer screening.

**Figure 1:**
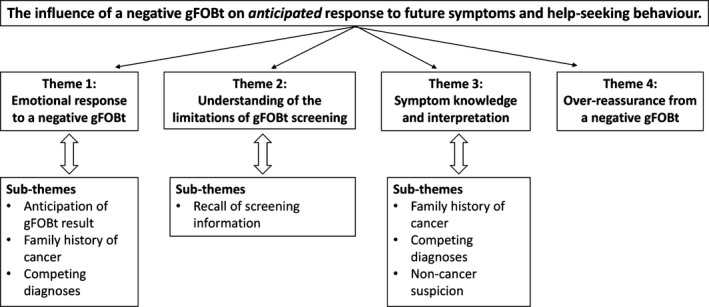
Identified themes and subthemes from the focus group analysis.

Four broad themes emerged from the focus group analysis, each with a number of influencing factors (subthemes): (i) *emotional* response to a negative gFOBt result (anticipation of test result, family history, competing diagnoses); (ii) understanding the limitations of gFOBt screening (recall of screening information); (iii) symptom knowledge and interpretation (family history, competing diagnoses and non‐cancer suspicion); and (iv) whether there was over‐reassurance from a negative gFOBt. Together these contributed to how participants understood a negative gFOBt and the impact on future symptom appraisal and help‐seeking behaviour. (See Fig. 1).

#### 3.2.1 Emotional response to a negative gFOBt result

Emotional response was influenced by three contributing factors (subthemes) including anticipation of their gFOBt result, for example whether or not they experienced any heightened anticipation or anxiety while awaiting their test result, family history of cancer and existing bowel concerns (competing diagnoses), for example irritable bowel syndrome (IBS).

In general, participants reported little, or no, apprehension while waiting to receive their result (normally within 2 weeks of completing their test kit), with some indicating that they had either forgotten about it or had “put it to the back of their mind”. However, often external influences such as televised cancer awareness campaigns would act as a trigger and remind them that they were still awaiting their screening result. Higher levels of apprehension were described by those who had been asked to do a repeat screening test (usually requested in response to an uncertain test result or damaged/incorrectly completed test kit) and by others who had a family history of bowel cancer. For participants with a family history of cancer, heightened apprehension tended to be most pronounced the first time that they took part in the screening programme.

…my dad actually had bowel cancer and had to have part of his bowel removed, and although that didn't kill him at the end, erm, that first, very first test when I sent it off, I was like a cat on hot bricks waiting for the result to come through.

(FG5: Female, Coventry)

Participants indicated that they felt a positive emotion such as “relief” or “pleasure” when they received their negative gFOBt result. Others described receiving a negative result to simply “meet their expectation” as they viewed themselves to be healthy, were not experiencing symptoms and therefore did not express strong positive or negative emotions in response to receiving a negative test result.

Of course, yes, you just think, oh, you've, you've escaped the noose this time.

(FG4: Male, Coventry)

…with not having problems I, I wasn't at all surprised when I got a negative result.

(FG2: Female, Coventry)

The feeling of relief or the reassurance felt having received a negative gFOBt result was more strongly conveyed in participants who had a family history of bowel cancer or who had other bowel concerns such as IBS.

The doctor did several tests and he just told me that it is IBS and there's nothing else for me to worry about. But, erm, I do like doing the bowel test screening, because I think it just puts your mind at rest, if there's bowel cancer or any type of cancer in the family, because it can come out in any way, shape or form.

(FG5: Female, Coventry)

#### 3.2.2 Understanding the limitations of gFOBt screening

A second broad theme to emerge was the extent to which participants understood or recognized the limitations associated with gFOBt screening. In part, this was dependent on their recall of the accompanying screening information provided with the screening test and result letter (subtheme). Participants were asked to think back to their negative (normal) test result letter and consider what they thought a negative result meant in terms of their CRC risk or the likelihood of developing symptoms in the future. When discussing the reliability of the test result, a number of participants indicated that they understood that screening was not a 100% guarantee that they did not have cancer and that they could not rely on the test result alone. Several participants recalled the disclaimer in the result letter that stated they should visit their general practitioner if they experienced particular symptoms listed in the letter.

There's a strong message that you don't rely on that [negative gFOBt result] and do nothing for the next two years…

(FG3: Male, Lothian)

However, a number of participants were surprised to discover that a negative gFOBt was not a 100% guarantee that they did not have cancer, with some participants stating that they did not always read the screening materials sent to them with their screening test or result letter—this was particularly evident for those who had taken part in screening over a number of years. For example, when the researcher read aloud the paragraph that accompanied the negative test result letter sent out in Coventry, England, which stated that no screening test is 100% effective and that a negative test does not guarantee that you do not have cancer or will never develop bowel cancer in the future, one participant replied,

…that didn't sink into me, I…you know, I wasn't really aware that, you know, things could still go wrong. It didn't come across to me too well, I just sort of sat there complacent thinking, you know, I'm okay.

(FG2: Female, Coventry)

When asked why they felt the test was not 100% effective (or reliable), participants typically raised issues of “human error” and the belief that “nothing is perfect”—rather than attributing incorrect results to either the features of the test or to tumour characteristics that can sometimes lead to a cancer being “missed”.

I'm not saying that, you know, there's lots of mistakes, but if you involve human beings, there's always the possibility of an error, nobody is perfect…I just know that no screening is perfect.

(FG2: Female, Coventry)

Screening results are based on a specific time‐point and may be different if screening is undertaken later. This temporal characteristic of a screening test result was recognized by some participants who indicated they understood that the test had not detected any signs of cancer at the time in which it was carried out, but recognized the possibility of “things going wrong” sometime thereafter. In three of the six focus groups, the time‐limited nature of the test result was described to be like a Ministry of Transport (MOT) test which is an annual test for vehicle safety carried out in the United Kingdom.

…it's like an MOT…it's valid on the day that the car gets done and after that things start falling off again.

(FG3: Male, Lothian)

#### 3.2.3 Symptom knowledge and interpretation

The impact of receiving a negative gFOBt result on anticipated (hypothetical) future symptom appraisal and help‐seeking behaviour was partly influenced by participant's existing knowledge of bowel cancer symptoms. The presence of a family history of cancer, competing bowel diagnoses and whether or not cancer was suspected contributed to how participants anticipated they might respond should they develop symptoms in the future. Participants unanimously felt that the introduction of screening programmes had increased their awareness of CRC. Those who had personal experience of family or friends with cancer communicated a greater depth of knowledge about the significance of symptoms—particularly the more common alarm symptoms associated with CRC. A number of participants recognized that the presence of blood in stools was a potential indicator of bowel cancer, and many stated that a change in regular bowel habits was, or could be, a possible symptom. Participants did not readily associate more vague symptoms such as tiredness or abdominal pain with symptoms of CRC, and despite having taken part in the screening programme, many for a number of years, a few participants disclosed that they felt they knew very little about the symptoms.

I wouldn't have a clue.

(FG1: Male, Coventry)

Personally I don't know what the symptoms are. I don't know…other than, other than blood, or certainly anything like that, but I've got no idea at all.

(FG5: Male, Coventry)

Participants recognized that competing bowel diagnoses such as IBS and haemorrhoids, and side‐effects of some long‐term medications, had the potential to mask possible cancer symptoms making symptom monitoring and appraisal more difficult.

I have irritable bowel, so I find it quite difficult to spot. Because if they say, spot any changes [in your bowel habits] mine changes on a daily basis.

(FG1: Female, Coventry)

Many participants had a positive attitude towards help‐seeking and agreed that they would exhibit prompt help‐seeking behaviours if they were to experience symptoms such as “blood in stool” or a change in their bowel movements. This was most strongly observed in participants with a family history of cancer. For less specific symptoms such as tiredness or abdominal pain, participants tended to describe more of a “watch and wait” self‐monitoring approach, and some participants indicated that they would not wish to waste the doctors’ time for symptoms that they perceived to be less serious, or that they did not readily associate with a potential cancer diagnosis. The symptom characteristics described to have the most influence on the decision to seek help (visit a GP) were a combination of a perceived “change from what was normal” and “symptom persistence” (rather than a “one‐off” symptom). It was also suggested by some participants that experienced symptoms may not be perceived as bowel cancer symptoms but rather something that was “unusual” or “uncomfortable” and which they would like to seek advice.

Well, you have a feeling yourself what's normal. And, I think if you notice a change that goes on, you know, perhaps the odd day it doesn't matter, but if it's something that goes over a week or, or so, then I think you probably start to think, you know, what it could be.

(FG2: Male, Coventry)

I think the symptoms that would take you to the doctor, you might not necessarily be thinking, bowel cancer, but, you know, if things in your ‐ for want of a better a word – plumbing start to go wrong, you might not necessarily think, I've got bowel cancer but you would go along, because it's…uncomfortable to live with if nothing else. Yeah.

(FG3: Male, Lothian)

#### 3.2.4 Whether there is over‐reassurance from a negative gFOBt result

The fourth theme to emerge from the data centred on the prospect of “over‐reassurance” following a negative gFOBt result and how taking part in CRC screening might inform the decision to visit a GP if participants later experienced symptoms. While many participants did not indicate that they felt that screening participation would influence their own help‐seeking behaviour, participants recognized that a negative gFOBt result had the *potential* to contribute to a delay in help‐seeking as a result of having been reassured by a recent negative result. Generally, any associated delay was attributed to how “others” might respond rather than how the participants themselves would respond, although some participants did indicate that they felt a negative gFOBt result might trigger a more optimistic approach in their own behaviour, leading them to potentially downplay symptoms or delay visiting their GP. Importantly, while participants felt that a negative gFOBt result might contribute to a delay in help‐seeking in the short term, while they monitored rather than ignored symptoms, they did not believe that it would deter them from going to their doctor if symptoms persisted.

…you'd probably be worried less, but there would come a point where you, you decide you have to go and get it looked at. So, it might, it might just sort of, er, influence you a bit [receipt of a negative FOBT]

(FG2: Male, Coventry)

I'd think well I've just had a test I'm okay. But, if..the symptoms did…carry on…, BUT I think I tend to be, er, more optimistic and wouldn't run to my doctors so quickly if I'd just had test results of negative.

(FG2: Female, Coventry)

## 4 Discussion

In this study, we explored how CRC screening participants understand and interpret a negative gFOBt result and whether receipt of a negative result has the potential to influence response to any future symptoms of CRC. There was variability in the extent to which a negative gFOBt result was interpreted as an “all clear”. While some participants acknowledged the residual risk of cancer associated with a negative gFOBt and the temporal nature of the test result, others were surprised to learn that a negative result was not a guarantee that they did not have cancer. Participants recognized that reassurance following a negative gFOBt had the *potential* to contribute to a delay in help‐seeking behaviour for symptoms suggestive of CRC, but overall participants did not feel that it would deter them from visiting their GP if symptoms persisted.

Many participants who were asymptomatic approached screening from the perspective that a negative result was mere confirmation of their health state while for others, particularly if there was a family history of bowel cancer or other bowel concerns such as IBS, a negative result was associated with positive emotions (e.g. relief). These findings suggest that despite acknowledging screening is “not perfect”, most participants experienced some level of reassurance from their negative result.

In colorectal and other cancer screening programmes, reassurance can be an important motivator, and a psychological benefit associated with participation.^22^, ^23^, ^24^ However, over‐reassurance can lead to negative consequences if, in turn, it influences a person's response, for example, by downplaying important symptoms. This was evident in the present study where some participants indicated that they felt their recent negative gFOBt result would lead them to be more optimistic and delay going to see their GP. There are some parallels with other forms of screening: among women diagnosed with an interval breast cancer, participation in mammography screening has the potential to lead to a “postponed reaction” to breast cancer symptoms.^19^ Similarly, over‐reassurance from a previous “all clear” or non‐cancer diagnosis following symptomatic presentation has been associated with delays in help‐seeking for possible cancer symptoms due to new symptoms being normalized or interpreted as benign.^25^ The way in which people recognize, interpret and respond to symptoms is complex and informed by a number of biopsychosocial, contextual and cultural influences,^17^ and therefore, reassurance or over‐reassurance from a negative gFOBt is likely to be just one of a number of potential factors that influence a person's decision to seek help for symptoms suggestive of CRC.

The study findings are consistent with a theoretical model of illness self‐regulation that proposes that people respond to symptoms using an iterative process governed by their representation of symptoms associated with particular illness labels and by their experience during a process of “lay hypothesis testing”.^26^, ^27^ The decision to consult a doctor might be more likely if people have a clear representation of the illness label associated with a particular symptom set and a clear representation of appropriate action. In common with previous work,^28^ the present study found that alarm symptoms or symptoms that were feared to be linked to cancer as well as symptoms that persisted over time were more likely to result in the decision to seek help. Furthermore, although not a prominent theme in the present study—perhaps because behaviour was anticipatory, the influence of the social context, for example the role of family members or carers, on symptom interpretation and help‐seeking behaviours could be further explored.

The results from the present study showed that some participants did not understand the nature of the limitations of gFOB screening in terms of the test or tumour characteristics^7^—the importance of which could be debated. Emphasis should be placed on the *temporal* nature of the test result with key messages around symptom recognition and advice about the benefits of prompt help‐seeking, particularly as Hall et al.^17^ reported that, for some participants, a recent negative screening result was associated with a reluctance to consult with bowel symptoms. Participants in this study did recognize the importance of continued participation in the screening programme. The analogy used by some participants, who described gFOB testing to be “like a car MOT”, could provide a more appropriate understanding of screening, acknowledging both the cyclical nature of screening and the time‐limited validity of the test result with the potential for things to still “go wrong” before the next screening round (or MOT) is due.

Participants who felt that their negative test result simply “met their expectations” attributed their response to a perception of “good health” and lack of experienced symptoms. In the screening literature, lack of symptoms has regularly been associated with lower perceived risk and lower uptake of cancer screening.^29^ This finding emphasizes the need for information that makes clear that the eligible population for cancer screening are asymptomatic individuals and that CRC can be diagnosed in those who perceive themselves to be fit and healthy.^30^

Family history of CRC and competing bowel diagnoses (primarily IBS in the present study, but potentially other bowel conditions such as Crohn's disease and haemorrhoids) were important moderators cross‐cutting a number of the study's findings. Studies have shown that family history contributes to perceived risk of cancer^31^ but has been reported to contribute less when family history is either “unknown” or where family members have little or no contact.^32^ Reassurance from a negative gFOBt, in the presence of a family history or a competing bowel diagnosis, was heightened among the participants of this study. Competing bowel diagnoses also had the potential to mask possible symptoms of CRC. The complexities around symptom monitoring and symptom appraisal among participants of CRC screening who have existing bowel complaints or other morbidities are not well documented and require further research.

It is important to note that this study explored participants’ anticipated response (or intended behaviour) to future symptoms of CRC. The gap between good intentions and behaviour is well recognized in the psychological literature,^33^, ^34^ and it is likely that participant's intentions might differ from their actual behaviour in the presence of real symptoms. Our study participants were almost exclusively from a white ethnic background, many were retired, and we found that participant attitudes towards screening were overwhelmingly positive. It is possible that participants may have been motivated to take part due to personal reasons that have increased their interest in this area or are more generally health aware than those who did not take up the invitation to attend a focus group. We did not have access to demographic information of those invited but who did not take part, to compare with our study sample.

It is also possible that there is some discrepancy between what was disclosed in a group setting (public persona) and what some individuals felt personally (private persona) that was not disclosed in the focus group discussions. This is a limitation of all focus group research.^35^ Similarly, participants’ opinions may have been influenced by the on‐going discussions and their personal opinions may have changed throughout the course of the focus group.

## 5 Conclusion

The importance of symptom vigilance between screening rounds is vital, given the limitations of gFOBt screening and the high incidence of interval cancers. Confidence in screening results has the potential to influence interpretation of symptoms and lead to a delay in help‐seeking. Effective early diagnosis and treatment might be further promoted by clear messages in information materials and result letters regarding the time‐limited value of the screening test and the nature of symptoms that should be reported to a GP. Further research might promote development of public health education materials that assist people in developing a mental representation of bowel symptoms. However, a major challenge for cancer screening programmes remains in finding new and innovative methods, that compliment on‐going initiatives in primary care in promoting symptom awareness and prompt help‐seeking behaviour, to deliver this information and engage with screening participants.

## Source of Funding

This study was funded by the National Awareness and Early Diagnosis Initiative led by Cancer Research UK, the Department of Health, NHS England and Public Health England. Award number C12357/A12240.

## Conflict of Interest

The authors declare that they have no conflict of interest.
